# Functional organization of motor networks in the lumbosacral spinal cord of non-human primates

**DOI:** 10.1038/s41598-019-49328-1

**Published:** 2019-09-19

**Authors:** Amirali Toossi, Dirk G. Everaert, Steve I. Perlmutter, Vivian K. Mushahwar

**Affiliations:** 1 Neuroscience and Mental Health Institute, University of Alberta, Edmonton, Alberta Canada; 2 Division of Physical Medicine and Rehabilitation, Department of Medicine, Faculty of Medicine and Dentistry, University of Alberta, Edmonton, AB Canada; 3 Department of Physiology and Biophysics, University of Washington, Seattle, Washington USA; 4 Washington National Primate Research Centre, Seattle, Washington USA; 5 Sensory Motor Adaptive Rehabilitation Technology (SMART) Network, University of Alberta, Edmonton, Alberta Canada; 6 Center for Sensorimotor Neural Engineering, Seattle, Washington USA

**Keywords:** Neural circuits, Spinal cord diseases

## Abstract

Implantable spinal-cord-neuroprostheses aiming to restore standing and walking after paralysis have been extensively studied in animal models (mainly cats) and have shown promising outcomes. This study aimed to take a critical step along the clinical translation path of these neuroprostheses, and investigated the organization of the neural networks targeted by these implants in a non-human primate. This was accomplished by advancing a microelectrode into various locations of the lumbar enlargement of the spinal cord, targeting the ventral horn of the gray matter. Microstimulation in these locations produced a variety of functional movements in the hindlimb. The resulting functional map of the spinal cord in monkeys was found to have a similar overall organization along the length of the spinal cord to that in cats. This suggests that the human spinal cord may also be organized similarly. The obtained spinal cord maps in monkeys provide important knowledge that will guide the very first testing of these implants in humans.

## Introduction

Recent advances in neuroprostheses have motivated a new wave of technologies aiming to augment the human body or restore its lost functions^1–3^. Neural networks of the spinal cord are one of the targets of these neuroprostheses for applications such as reanimating paralyzed limbs^4–9^, reducing mobility deficits^10^, and promoting targeted plasticity and recovery^11^ after neural injury and disease.

An example of these neuroprostheses is the intraspinal microstimulation (ISMS) implant which is comprised of an array of ultra-fine electrodes that deliver electrical pulses to the ventral horns of the spinal cord^12^. ISMS can produce functional movements of the lower and upper limbs (lumbosacral and cervical implants)^5,9^, breathing (cervical implant)^13^ or bladder function (sacral implant)^14^ depending on the targeted region within the spinal cord. Stimulation through an individual intraspinal electrode can activate motor networks including motoneurons, afferent^15^ and propriospinal^16^ projections, and associated axons that span multiple spinal cord segments^15^. A small number of implanted electrodes can evoke synergistic muscle contractions and produce coordinated movements involving single or multiple joints that can perform functional tasks^4,17–19^.

ISMS in the lumbosacral spinal cord has been widely studied and has shown promising results in animals for the restoration of hindlimb movements after paralysis. Notably, hindlimb movements evoked by ISMS in cats were significantly more fatigue resistant compared to those obtained by intramuscular electrical stimulation^4,18^. With ISMS implants, animals could stand for ~5x longer durations^18^ and walk over-ground for ~10x longer distances^4^ than animals with intramuscular implants.

The cat has been the classical model for ISMS research in the lumbosacral spinal cord. Placement of ISMS electrode arrays in this species is guided by knowledge of the functional organization of the motor networks in the spinal cord^5,20,21^. This knowledge was derived from investigations of the movements evoked by ISMS in various parts of the ventral horn along the length of the lumbosacral enlargement, which led to the formation of a functional map^20^. Numerous studies of ISMS in cats have shown that the functional map is consistent between animals^4–6,18,20–22^.

One requirement for translating ISMS to clinical implementation is gaining knowledge about the functional organization of the motor networks to be targeted in the lumbar spinal cord of humans. Information regarding the anatomical organization of the motoneuronal cell bodies in the human lumbar spinal cord that innervate the leg muscles (i.e., anatomical map) exists^23–25^. However, unlike in cats, the functional organization and connectivity of various motoneuronal pools (i.e., functional map), and the required stimulation amplitudes for their activation, are not known. In this study, we took a critical step towards answering these questions and investigated the functional map of the lumbar spinal cord in another primate, the macaque monkey. Based on the evidence from functional mapping experiments in various mammals (cats^20,22^, rats^26–29^ and pigs^30^), we hypothesized that a similar functional organization of motor networks is preserved in non-human primates. This study allowed us, for the first time, to identify empirically the functional connectivity of motor networks in the primate lumbosacral cord with a high spatial resolution.

## Results

### Functional map of the lumbosacral spinal cord

**Figure 1:**
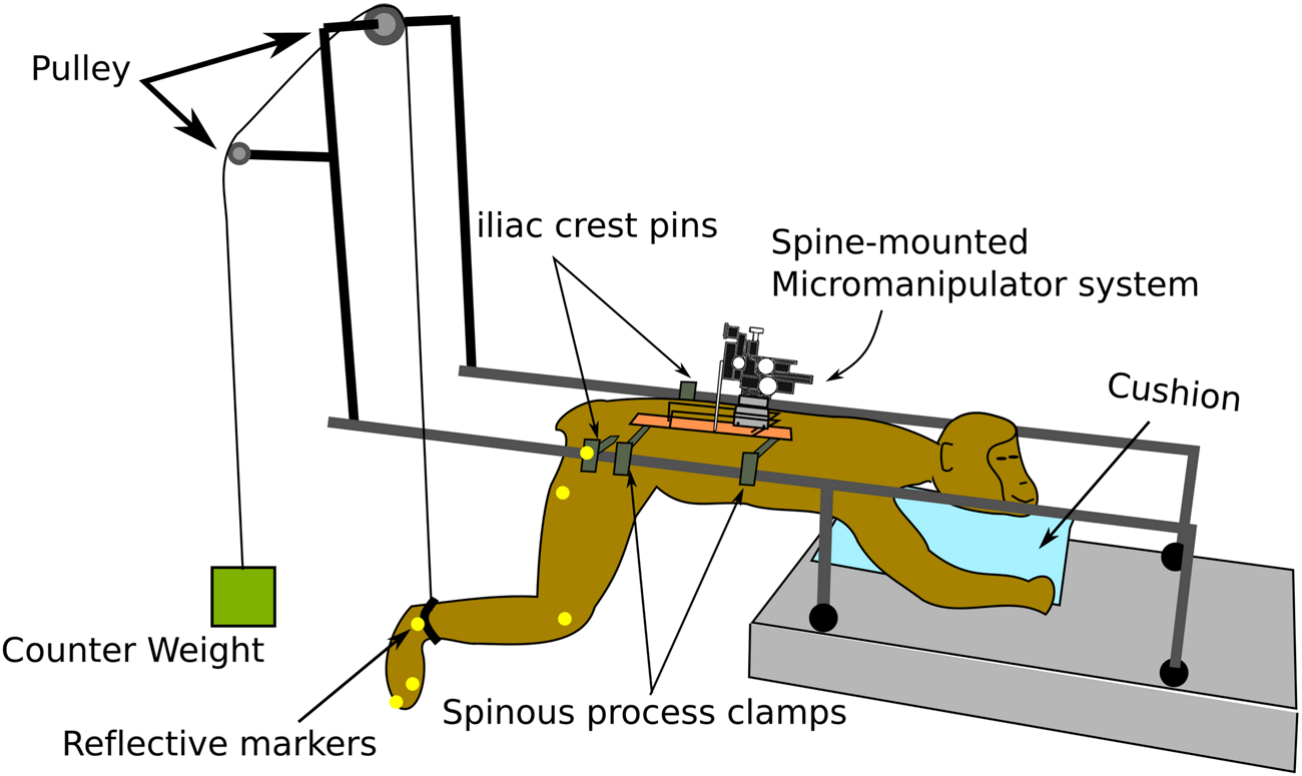
Experimental setup for functional mapping of the lumbosacral spinal cord in non-human primates.

Experiments were conducted on 4 skeletally-mature rhesus macaque monkeys (three females and one male, 9.3 ± 1.8 kg) under pentobarbital anesthesia. Responses to intraspinal microstimulation under pentobarbital anesthesia have previously been shown to be representative of those evoked in awake animals^5,6^. The lumbosacral enlargement of the spinal cord was surgically exposed and a stereotactic setup with a micromanipulator was assembled onto the spine^31^. The micromanipulator was used to guide a microelectrode to various locations in the ventral horn of the gray matter. Each of the locations was stimulated with current amplitudes up to 120 µA. If no movement was evoked with currents up to this amplitude, the micromanipulator was advanced to a new location. For all locations where movements were produced, movement type and stimulation threshold were recorded. At select locations, kinematics, joint torques, and electromyographic (EMG) activity were also recorded. In order to visualize and quantify the evoked movements, the hindlimb ipsilateral to the stimulated side of the spinal cord was suspended through a pulley system as shown in Fig. 1.

**Figure 2:**
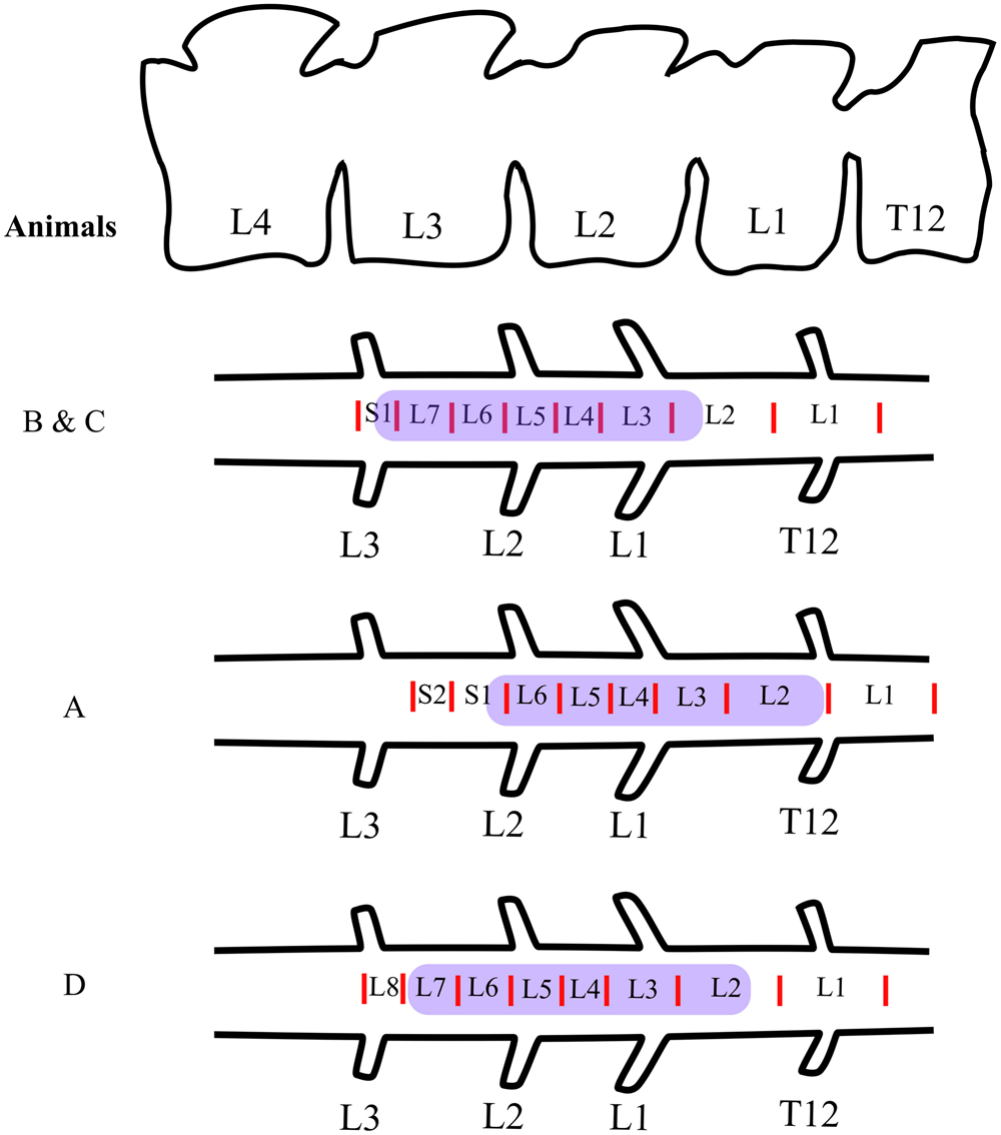
Anatomical location of the mapped region of the spinal cord in this study (highlighted in purple). For all animals, the spinal cord segments were identified based on the entry zones of the dorsal rootlets.

The lumbosacral enlargement of the spinal cord spanned an approximately 4 cm-long region. Two animals (B and C in Fig. S1) had 7 lumbar vertebrae and spinal cord segments, one animal (A) had 6 lumbar vertebrae and spinal segments and one animal (D) had 8. The lumbosacral enlargement contained spinal segments L3-S1, L2-L6, and L3-L8 for animals with 7 (most common anatomy^32–34^), 6 and 8 lumbar vertebrae, respectively. This region was located within vertebral levels L2-L3 for animals with 6 lumbar vertebrae, and L1-L3 for animals with 7 and 8 lumbar vertebrae (Fig. 2). The entire length of the lumbosacral enlargement was mapped in monkeys A, B, and D, and partially mapped in monkey C (L4 and L5 were not mapped in this animal).

**Figure 3:**
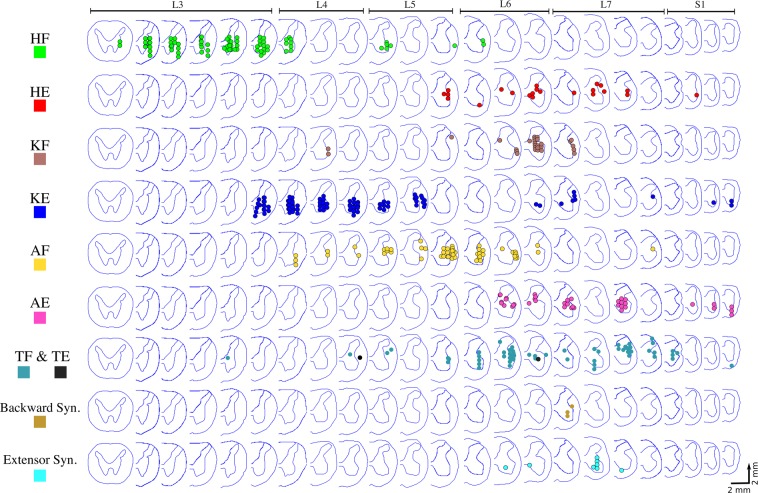
Functional map of the lumbosacral enlargement of the spinal cord acquired from 4 rhesus monkeys. ISMS in the locations shown on the maps evoked movements with thresholds ≤120 µA. Each dot represents a mapped location in the spinal cord that produced a movement. Different colors represent different movements. Spinal cord cross-sections shown in each row are 2 mm apart from their neighboring cross-section, irrespective of their spacing in the figure. Total length of the spinal cord covered by the cross sections shown is ~46 mm. HF: Hip Flexion, HE: Hip Extension, KF: Knee Flexion, KE: Knee Extension, AF: Ankle Flexion, AE: Ankle Extension, TF: Toe Flexion, TE: Toe Extension, Backward syn.: Backward Synergy (HE + KF + AE), Extensor Syn.: Extensor Synergy (HE + KE + AE).

The functional maps constructed from all animals are shown in Fig. 3. Alignment of the spinal cords between animals was based on the morphology of the ventral horns. Studies of the anatomical and functional organization of motoneuronal pools in the feline lumbosacral enlargement suggest that the morphology of the ventral horns is the best indicator of motoneuronal and functional organization^20,35^. Dots in the figure show sites where movements were evoked by ISMS. Spinal cord sections that do not show any colored dots were not mapped. All mapped locations, including those that did not produce movements with current amplitudes up to 120 µA, are shown in Fig. S1. In all animals, stimulation in segments rostral to the lumbosacral enlargement evoked paraspinal muscle contractions while stimulation in segments caudal to the enlargement evoked tail movements.

In total, 697 locations were stimulated in the spinal cords across all animals, of which 56% (390) evoked a movement. Stimulation in the most rostral quarter of the enlargement (~1 cm long) predominantly evoked hip flexion. Caudal to this region, a region ~1.6 cm-long, produced knee extension, hip adduction and ankle flexion (dorsiflexion). Locations evoking ankle flexion were found laterally to locations that evoked knee extension. The remaining caudal third of the enlargement (~1.4 cm long) evoked more diversified movements including single joint movements such as ankle extension (plantar flexion), knee flexion, toe flexion and extension, and multi-joint synergistic movements including extensor and backward synergies. An extensor synergy was defined as the combination of hip, knee, and ankle extension. A backward synergy was defined as the combination of hip extension, knee flexion, and ankle extension.

**Figure 4:**
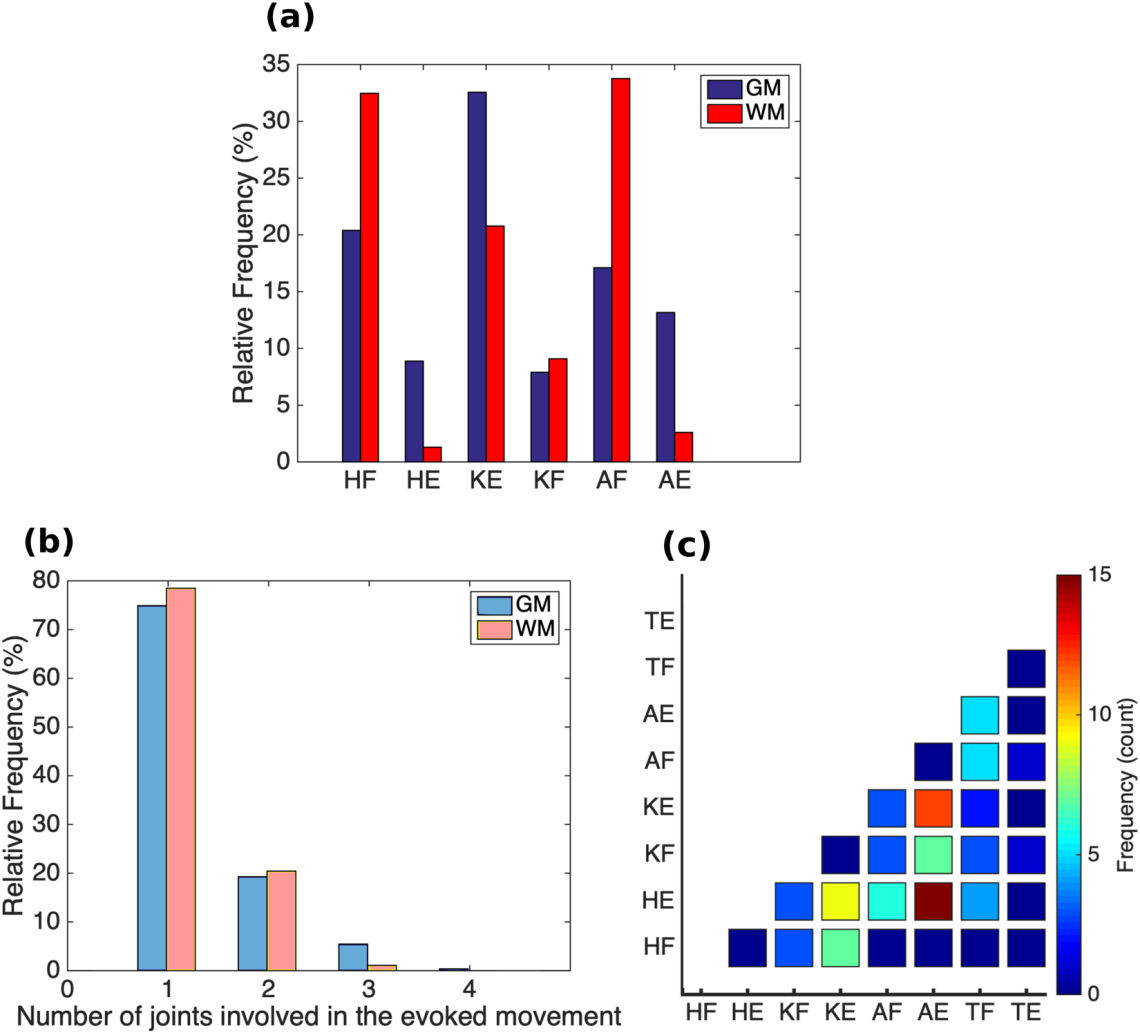
(**a**) Overall distribution of the main leg movements resulting from microstimulation in the gray matter (GM) and white matter (WM) of the lumbosacral spinal cord in all animals (n = 4). **(b)** Number of joints (hip, knee, ankle and, metatarsophalangeal (MTP)) involved in all evoked leg movements. **(c)** Distribution and frequency (count) of the multi-joint movements evoked by microstimulation only in the GM in all animals. HF: Hip Flexion, HE: Hip Extension, KF: Knee Flexion, KE: Knee Extension, AF: Ankle Flexion, AE: Ankle Extension, TF: Toe Flexion, TE: Toe Extension.

Distribution of the main evoked movements is shown in Fig. 4a. Knee extension was the most common movement (32.6%), followed by hip flexion (20.3%). Almost a quarter of the evoked movements (89/390) involved more than one joint (Fig. 4b). Most of these movements were evoked by ISMS in the caudal third of the enlargement. Multi-joint movements were more commonly combinations of joint extension than flexion movements (Fig. 4c). The most common multi-joint movement was hip and ankle extension (15/390), followed by knee and ankle extension (12/390).

### Stimulation thresholds

**Figure 5:**
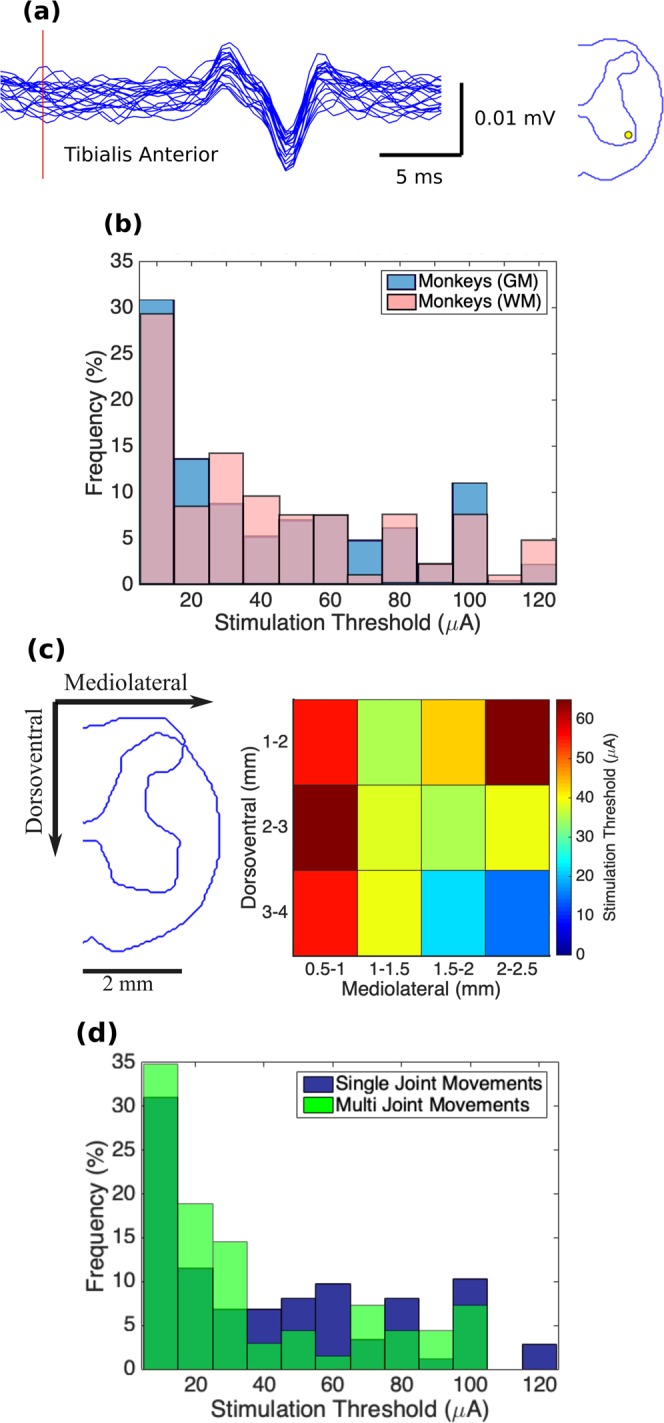
(**a**) Example of EMG activity evoked in the tibialis anterior muscle by ISMS at threshold intensity (10 μA) in Monkey D. Red vertical line represents stimulation onset. Figure on the right shows the location of the electrode tip on a transverse cross-section of the spinal cord. **(b)** Distribution of the stimulation thresholds for producing leg movements in the gray (GM) and white (WM) matters (n_GM_ = 227 and n_WM_ = 106) of the spinal cord (n = 4 animals). **(c)** Spatial distribution of the stimulation thresholds. Colors in each bin represent the mean threshold across all animals. Mediolateral coordinates are shown with respect to midline. Dorsoventral coordinates are shown with respect to the dorsal surface of the spinal cord. **(d)** Comparison of the stimulation thresholds for single- and multi-joint movements evoked by ISMS. Distribution of the stimulation thresholds for single-joint (n = 174) and multi-joint (n = 69) movements evoked by ISMS in the spinal cord of 4 animals.

Stimulation threshold was defined as the lowest ISMS amplitude required to evoke a visible twitch or movement in the targeted muscles (Fig. 5a). The stimulator used for this study provided stimulation amplitudes as low as 10 µA, but not lower. The largest proportion of the stimulation thresholds were ≤10 µA (Fig. 5b). This was the case for locations stimulated in both the gray matter (31%) and white matter (29%) of the spinal cord. Moreover, 65.6% of the locations in the gray matter and 68.9% in the white matter had stimulation thresholds ≤50 µA. The average stimulation threshold was lower for more ventral and more lateral locations in the transverse plane of the spinal cord (Fig. 5c). No statistical difference was found between the stimulation thresholds for evoking single- and multi-joint movements (p = 0.09, Fig. 5d).

### Kinematics

**Figure 6:**
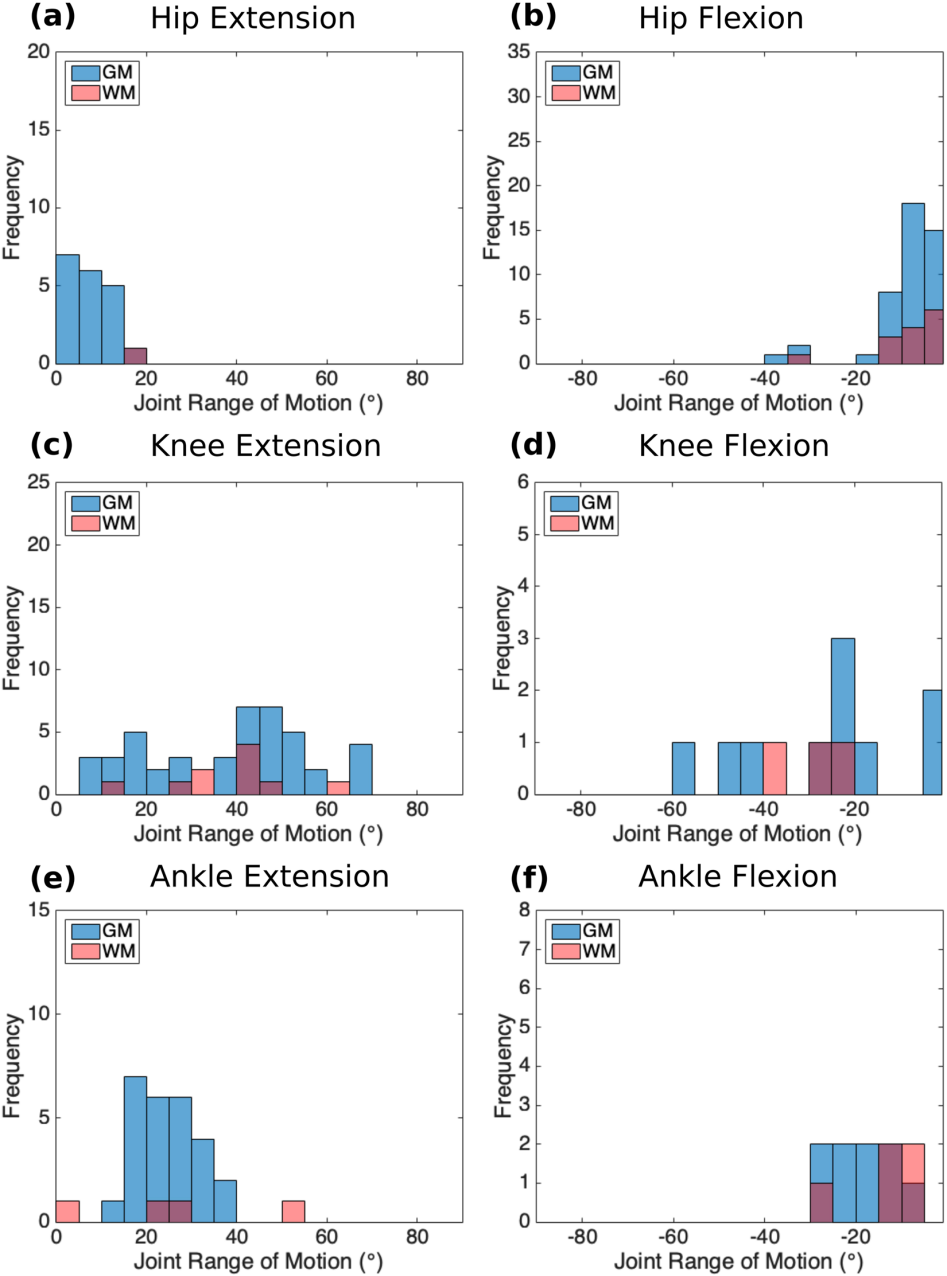
Distribution of changes in joint angle (i.e., range of motion) for the movements evoked by ISMS at sites in the gray and white matter (GM and WM) in all animals. Kinematic measurements were obtained for 40% of all sites with ISMS-evoked movements. **(a)** Hip Extension (initial hip angle: 114.6° ± 3.2° [mean ± standard error]). **(b)** Hip Flexion (initial hip angle: 112.6° ± 3.3° [mean ± standard error]). **(c)** Knee Extension (initial knee angle: 84.8° ± 3.86° [mean ± standard error]). **(d)** Knee Flexion (initial knee angle: 102.9° ± 7.9° [mean ± standard error]). **(e)** Ankle Extension (initial ankle angle: 116.1° ± 3.9° [mean ± standard error]). **(f)** Ankle Flexion (initial ankle angle: 114.5° ± 5.4° [mean ± standard error]).

**Figure 7:**
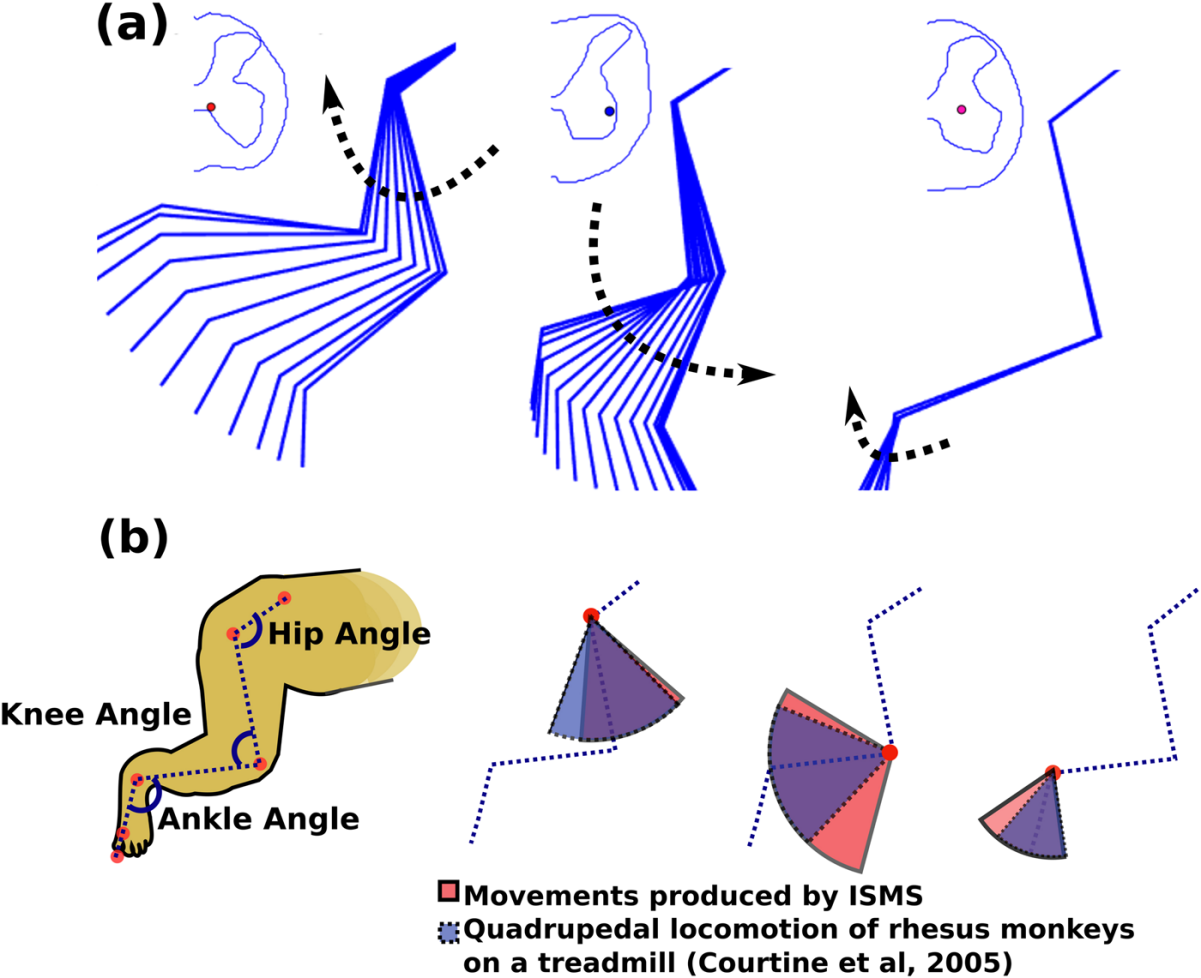
**(a)** Examples of the ISMS-evoked range of motions in the ipsilateral hindlimb of animal A. The location of the electrode tip for each movement is shown on the cross-sectional trace of the spinal cord. **(b)** Range of motion (ROM) of hip, knee and ankle joints produced by ISMS in the gray matter of the spinal cord (in Red). ROM of hip, knee and ankle joints of rhesus monkeys during quadrupedal locomotion on a treadmill at a speed of 1.79 m/s (in Blue). Treadmill locomotion data were obtained from Courtine *et al*.^36^.

Hindlimb movements were recorded in the sagittal plane with a single camera. The pelvis was fixed in a frame at the iliac crests. Free movement of the hip, knee and ankle joints was achieved by suspending the leg with a counter weight attached through a pulley system to a strap fitted above the ankle joint (Fig. 1). In total, kinematic recordings were obtained from 40% of the ISMS sites that evoked responses. For these measurements, the spinal cord was stimulated at a current amplitude beyond which joint range of motion (ROM) did not increase (up to a maximal limit of 300 µA). The distribution of changes in the hip, knee and ankle joint angles in response to ISMS in various locations of the gray and white matter is shown in Figs 6 and S2. Movements of the knee and ankle joints were larger than movements of the hip. The evoked ROM for hip flexion and extension movements was 8.68° ± 7.71° (mean ± standard deviation) and 8.18° ± 5.27°, respectively. The evoked ROM for knee flexion and extension movements was 26.98° ± 16.63° and 37.8° ± 17.17°, respectively. The evoked ROM for ankle flexion and extension movements was 15.16° ± 10.55° and 23.1° ± 13.74°, respectively. The largest total ROM (the combined flexion and extension ROM of a joint) evoked by ISMS for the hip, knee and ankle joints was 52°, 107° and 65°, respectively (Fig. 7).

The total ROMs of the hip, knee and ankle joints during movements evoked by ISMS were compared to those produced during quadrupedal locomotion on a treadmill^36^. Fig. 7 shows the measured ROMs while stepping on a treadmill belt moving at a speed of 1.79 m/s and the total ROMs evoked by ISMS in this study. The hip ROM induced by ISMS was 22% smaller (in extension) than the ROM during treadmill locomotion, while the ISMS-induced ROMs were larger at the knee (by 35%) and ankle (by 30%) joints.

### Torque

Isometric forces were measured while isolating the evoked movement to a single joint. Similar to the kinematic measurements, forces were recorded with the spinal cord stimulated at a current amplitude beyond which the joint ROM did not increase (maximal limit of 300 µA). In total, forces were recorded for 31/390 locations in the spinal cord that evoked a movement, 27 of which were in the gray matter and 4 in the white matter. The forces were converted to joint torques based on the moment arm. The distribution of all torque measurements over the lumbosacral enlargement is shown in Fig. S3.

**Figure 8:**
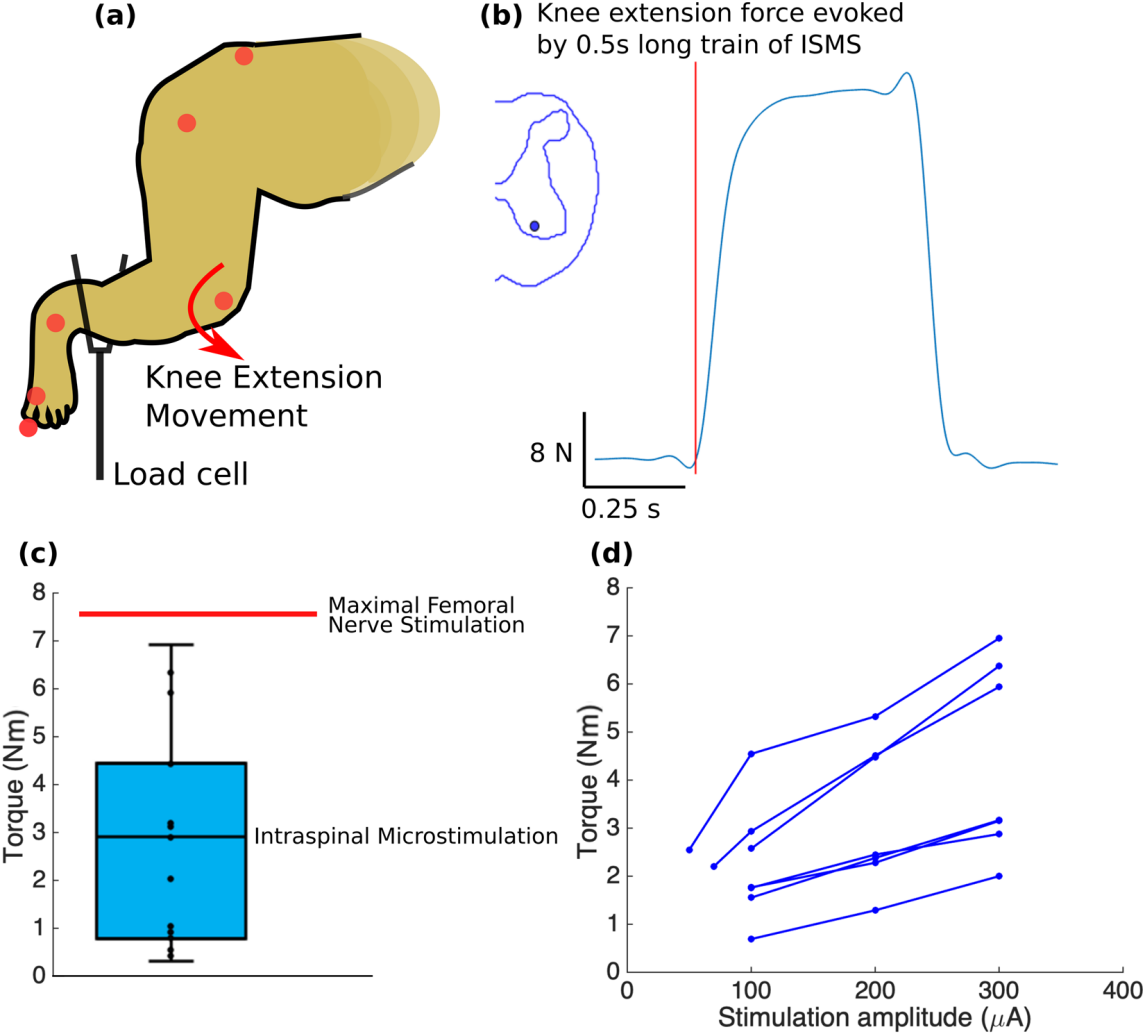
**(a)** Isometric torque measurement setup. **(b)** Example of a force trace for an ISMS-evoked knee extension movement, recorded using the load cell. **(c)** Isometric torque measurements for knee extension movements evoked at 15 select locations across 4 animals. Whiskers show the minimal and maximal values and box represents the interquartile range. Moment arm was 15.9 ± 1.1 cm (average ± standard deviation). Knee extension torques produced with ISMS in the gray matter of the spinal cord (in cyan) and with femoral nerve stimulation (n = 1 recording - red horizontal line) are shown. **(d)** Knee extension torque recruitment curves recorded for 7 locations in the gray matter of the spinal cord in animals B and C. The stimulation protocol consisted of 0.5 s-train of biphasic, charge-balanced pulses, 200 µs-long and delivered at 50 Hz frequency.

Knee extension torques measured for 17 select locations (Fig. 8a–c) ranged from 0.25 to 6.95 Nm with a moment arm of 15.9 ± 1.1 cm. For comparison, in one experiment (animal A), a nerve cuff was placed around the femoral nerve and stimulated supra-maximally. The recorded torque under this condition was 7.6 N.m. Therefore, ISMS through a single electrode produced up to 91% of the maximal possible torque.

Ankle extension torques measured in 3 select locations ranged from 0.23 to 2.02 Nm (moment arm: 7.6 ± 1.2 cm). Ankle extension torques resulting from supra-maximal stimulation of the tibial nerve were also recorded (n = 3, animals A, B, and D) and ranged from 5.9 to 8.5 Nm. ISMS through a single electrode therefore produced 23.8 to 34.5% of the maximal possible torque.

Measured hip flexion torques (n = 3) ranged from 0.26 to 1.21 Nm (moment arm: 15.3 ± 1.1 cm). Isometric forces were also measured for ISMS-evoked extensor synergies (n = 3) and ranged from 6.9 N to 46.1 N.

Examples of torque recruitment curves recorded for 7 ISMS locations producing knee extension are presented in Fig. 8d. These curves show that by increasing stimulation intensity, joint torques increase gradually, in contrast with the steep increases in torque for small increases in stimulus amplitude often associated with peripheral nerve stimulation. This finding is consistent with studies of ISMS in cats^22,37^ and rats^26^.

### Muscle activity

EMG activity resulting from microstimulation in the spinal cord was recorded through pairs of intramuscular wire electrodes placed in 8 muscles: vastus lateralis, vastus medialis, tibialis anterior, medial gastrocnemius, lateral gastrocnemius, sartorius, semimembranosus anterior, and biceps femoris posterior. EMG recordings were used in conjunction with kinematic recordings to identify the types of evoked movements, and distinguish passive (driven by gravity) and active movements.

**Figure 9:**
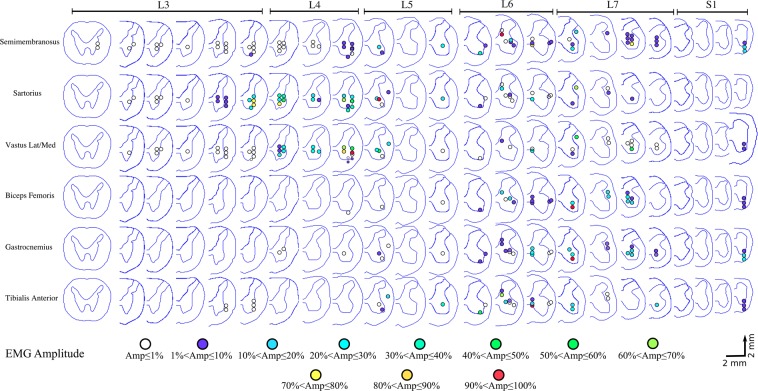
Spatial distribution of the mapped locations that evoked muscle activity in the semimembranosus, sartorius, vastus lateralis and medialis, biceps femoris, gastrocnemius, and tibialis anterior muscles of the rhesus monkey. Results are obtained from electromyography (EMG) recordings from animals A, C, and D. Stimulation amplitude was 100 µA for all recordings. Amplitudes of the EMG signals recorded from each muscle in each animal were normalized to the maximal amplitude recorded for that muscle in that animal.

Figure 9 summarizes the evoked EMG activity by ISMS across the lumbosacral enlargement. Included in this map are all recorded trials in animals A, C and D where ISMS was delivered in the gray matter and evoked a movement with a stimulation amplitude of 100 µA. Semimembranosus, sartorius, and vastus muscles were activated by stimulation in most spinal segments of the lumbosacral enlargement (L3–L7). In contrast, biceps femoris, gastrocnemius, and tibialis anterior muscles were predominantly activated by stimulation in more caudal spinal segments of the enlargement (L5–L7). In spinal segments L3–L5, sartorius and vastus muscles had a similar activation pattern (active together in 35/36 sites) with the largest responses evoked by stimulation in the L4 and L5 segments. In spinal segment L6, gastrocnemius-tibialis anterior, semimembranosus-sartorius, gastrocnemius-tibialis anterior-biceps femoris muscle groups showed a close activation pattern: active together in 12/13, 13/13, and 10/13 sites, respectively. In spinal segments L6–L7, semimembranosus-gastrocnemius muscles were active together in 23/26 sites.

## Discussion

### Functional map of the lumbar enlargement of the spinal cord

The functional maps obtained in this study were acquired under pentobarbital anesthesia. Previous ISMS results from spinal cord injured and intact animals^5,6,38^ suggest that responses evoked under this anesthetic agent are representative of those evoked in awake animals. In an awake animal with an intact spinal cord, responses to ISMS may be modulated by sensory or descending inputs. Anesthetized animals are a suitable model of a spinal cord with a complete injury and diminished descending input, and provide a relevant model for testing of ISMS since that the primary intended purpose of ISMS implants is to restore mobility after severe spinal cord injury (SCI).

Functional studies of ISMS for restoring standing and walking have been mainly conducted in medium to small animal models (cats^4–6,18,20,21^ and rats^26^,^27^). Although anecdotal testing has been conducted in large animal models (pigs^30^), cross species comparisons of the functional networks targeted by ISMS has not been performed. The maps obtained in this study are the first high resolution functional maps of the lumbosacral spinal cord in primates. These maps demonstrate that organized, compact motor networks exist in the lumbosacral spinal cord of rhesus monkeys which may be targeted to restore lower limb mobility. Despite the natural variability of the number of lumbar vertebral segments in the studied animals^33,34^, the relative organization of the motor networks in the obtained functional maps was consistent from one animal to another. In all animals, hip flexors were activated in more rostral regions of the lumbar enlargement than knee extensors, followed by ankle flexors, hip extensors, toe flexors, ankle extensors, extensor synergy, knee flexors, and backward synergy. A consistent organization was also observed in the mediolateral and dorsoventral dimension where, for instance, ankle flexors were evoked at more lateral and more dorsal locations in the gray matter than knee extensors. Hip extensors were also evoked at more dorsal locations than the knee extensors.

**Figure 10:**
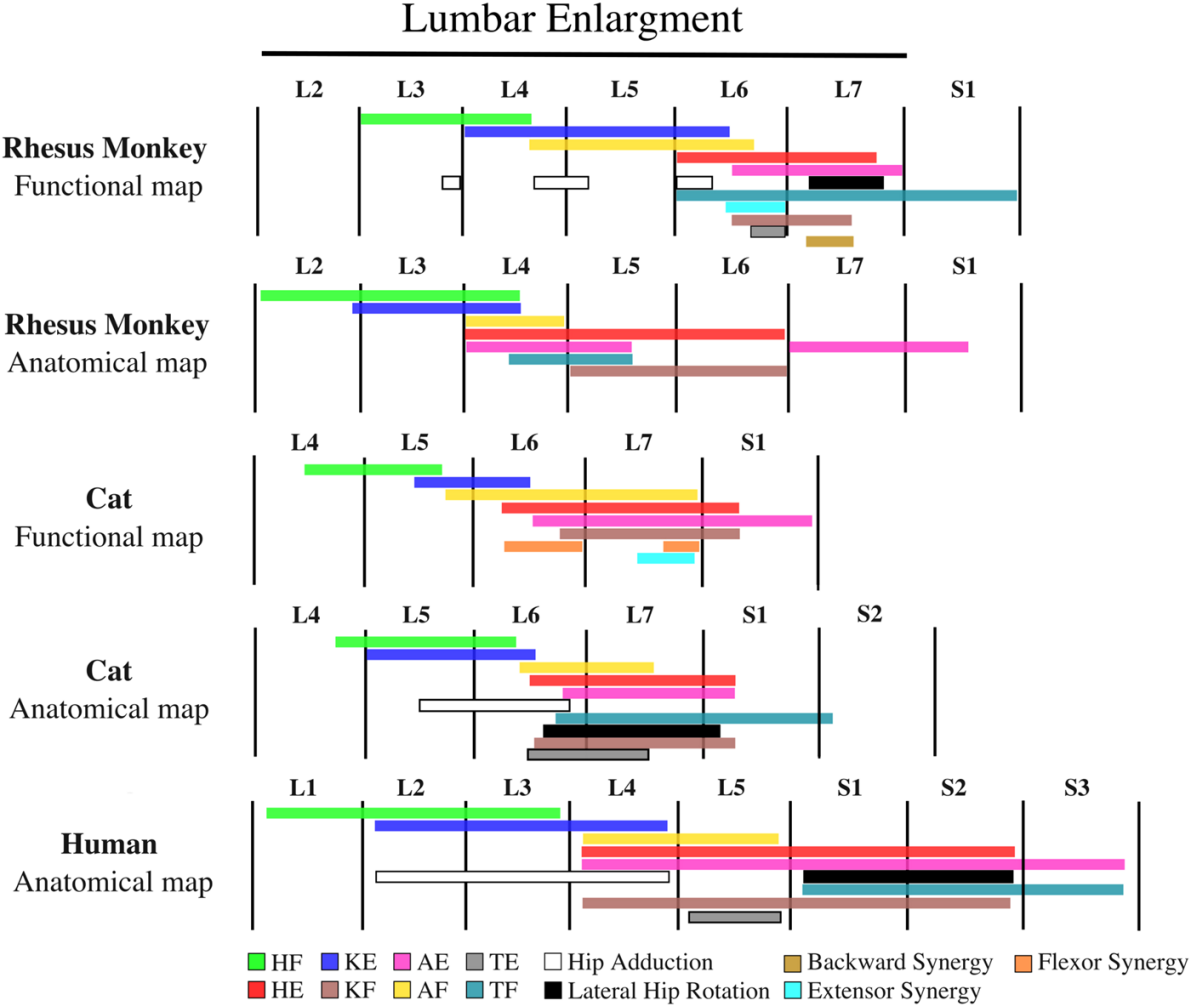
Rostrocaudal organization of the lumbar enlargement of the spinal cords of rhesus monkeys, cats and humans. In all species, hip flexors were activated in more rostral regions of the lumbar enlargement than knee extensors, followed by ankle flexors, hip extensors, ankle extensors and knee flexors. Data for the monkey anatomical map were obtained from Capogrosso *et al*.^10^. Data for the human anatomical map were obtained from Sharrard^23,24^. The resolution of the human spinal cord anatomical map is limited to full spinal cord segments. Data for the cat functional map were obtained from Mushahwar *et al*.^21^, Saigal *et al*.^5^, and Mushahwar & Horch^20^. Data for the cat anatomical map were obtained from Vanderhorst & Holstege^35^, and Yakovenko *et al*.^39^. The total length of the lumbar enlargement in monkeys, cats and humans are approximately 40 mm, 30 mm^6,20,55^ and 50 mm^56^, respectively. Extensor synergy is defined as a combination of HE, KE, and AE and backward synergy is defined as HE, KF, and AE. HF: Hip Flexion, HE: Hip Extension, KF: Knee Flexion, KE: Knee Extension, AF: Ankle Flexion, AE: Ankle Extension, TF: Toe Flexion, TE: Toe Extension. Anatomical map of the cat lumbosacral spinal cord was adapted based on the following motoneuronal pools: HF – Psoas, Sartorius, Iliacus, Rectus Femoris, Gracilis; HE – Semimembranosus, Semitendinosus, Biceps Femoris, Gluteus maximus; Hip Adduction – Pectineus, Adductor Femoris Magnus, Gracilis, Adductor brevis, Adductor longus; Lateral Hip Rotation – Gluteus maximus, Internal obturator; KE – Rectus Femoris, Vastus Medialis, Vastus Lateralis, Vastus Intermedius;, KF – Biceps Femoris, Semitendinosus, Semimembranosus; AF – Extensor digitorum longus, Tibialis anterior; AE – Flexor halluces longus, Tibialis posterior, Plantaris, Soleus, Lateral and Medial Gastrocnemius; TE – Extensor digitorum longus muscle; TF – Intrinsic foot, Flexor hallucis longus, Flexor digitorum longus. Muscles that were used for generating the anatomical map for humans were: Psoas, Hip adductors, Quadriceps, Sartorius, Tibialis anterior, Extensor digitorum longus, Tibialis posterior, Knee flexors, Gastrocnemius, Soleus, Peroneus, Intrinsic foot, Flexor digitorum longus, Gluteus maximus, and Lateral hip rotators. Anatomical map of the monkey lumbosacral spinal cord was adapted based on the following motoneuronal pools: HF – Psoas, Rectus femoris; HE – Gluteas medius, Semitendinosus; KE – Rectus femoris; KF – Semitendinosus; AF – Tibialis anterior, Extensor digitorum longus; AE – Medial Gastrocnemius, Flexor digitorum longus; TF – Flexor Digitorum Longus.

A Comparison of the functional map of the lumbosacral enlargement obtained in this study with a coarse anatomical map for rhesus monkeys^10^ suggests a similar relative organization between the motor networks in the rostrocaudal direction (Fig. 10). This comparison will likely be further enhanced should more complete anatomical maps become available in the future. The anatomical map of the lumbosacral enlargement in Fig. 10 was adapted from Capogrosso *et al*.^10^ While, to the best of our knowledge, this is the only anatomical map of the lumbosacral enlargement available in rhesus monkeys, it represents a small number of muscles acquired from a limited number of animals (12 muscles, each investigated in 1 or 2 animals), and major muscles were omitted. For instance, knee extensors represented in this map are based on the rectus femoris motoneuronal pool, and does not include pools innervating the vastus muscles. Similarly, major hip extensors (the semimembranosus, biceps femoris, and gluteus maximus), hip flexors (sartorius, gracilis, and iliacus), knee flexors (biceps femoris and semimembranosus muscles), and ankle extensors (soleus and lateral gastrocnemius) are not represented in the anatomical map. Nevertheless, this map provides a valuable insight into the organization of the motoneuronal pools in monkeys.

**Figure 11:**
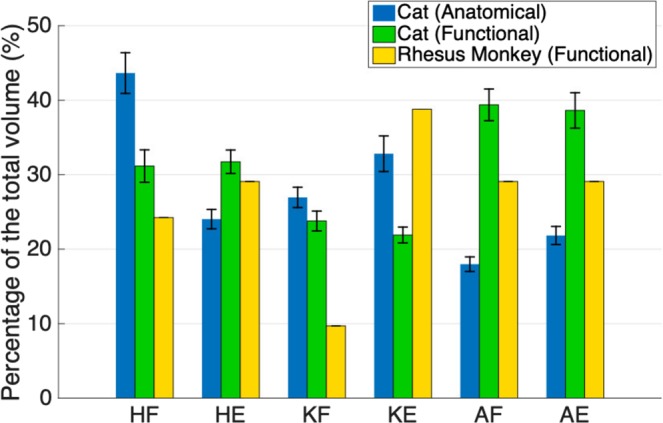
Spatial distribution (in the rostrocaudal direction) of the maps of the lumbar enlargement in cats and rhesus monkeys. The total length of the lumbar enlargement in the spinal cords of monkeys, cats and humans are approximately 40 mm, 30 mm^6,20,55^ and 50 mm^56^, respectively. Functional map data for cats were obtained from Mushahwar & Horch^20^, Saigal *et al*.^5^, and Mushahwar *et al*.^21^. Anatomical map data were obtained from Vanderhorst & Holstege^35^. Functional maps only include responses from ISMS in the gray matter of the spinal cords. The sizes of the spinal cord segments in cats used to convert the maps into spatial distribution were derived from measurements obtained from 4 cat spinal cords. HF: Hip Flexion, HE: Hip Extension, KF: Knee Flexion, KE: Knee Extension, AF: Ankle Flexion, AE: Ankle Extension. Error bars represent standard deviation.

To investigate possible trends in the functional organization of the mammalian lumbosacral spinal cord across species and extrapolate to the human spinal cord, comparisons were made between the functional maps obtained in monkeys and existing maps in cats^5,20,21^. As shown in Fig. 10, the rostrocaudal organization in cats and monkeys is similar. A similar relative organization is also seen for ankle flexors and knee extensors in the mediolateral and dorsoventral dimensions^20^. The spatial distribution of the functional elements of the maps in cats and monkeys is shown in Fig. 11. Despite the similar organization of the functional maps, knee extension is evoked in a larger portion of the enlargement in monkeys (39%) compared to cats (22%). Knee flexion however, is evoked in a smaller portion of the enlargement in monkeys (10%) compared to cats (24%).

In addition to the functional organization of the lumbosacral motoneuronal pools, the anatomical location of the motoneuronal cell bodies innervating leg muscles (i.e., the anatomical organization of the motoneuronal pools) are also known in cats^35,39^ (Fig. 10). A comparison between the anatomical and functional maps in cats reveals a similar relative rostrocaudal organization in both maps.

While the functional organization of the lumbosacral motoneuronal pools is not known in humans, the anatomical organization of the motoneuronal pools in this region is available from Sharrard’s studies in polio patients^23,24^. The myotomal innervation of the leg muscles is also known in humans^25^ and has a similar distribution as the anatomical maps described by Sharrard^23,24^. As shown in Fig. 10, the anatomical organization of the motoneuronal pools in the human spinal cord is also similar to the functional and anatomical maps in monkeys and cats.

Given the similarity of the relative organization of the motor interneuronal networks across species, and the similarity of the organization of the anatomical and functional maps in cats and monkeys, we hypothesize that the human functional map will also have the same organization.

It is important to consider that while rhesus monkeys commonly ambulate quadrupedally, their walking is different from that of the sub-primate animals^36^. Characteristics such as the diagonal footfall pattern and the differences in the recruitment pattern of the distal muscles of the hind- and forelimbs distinguish the rhesus monkeys from cats^36^. These differences in the muscle recruitment patterns may have led to the observed differences in the spatial distribution of the functional maps in cats and monkeys. Bipedal walking in humans involves differences in the muscle recruitment patterns of the legs compared to quadrupedal walking in monkeys and cats^40–43^. Since differences exist between the neuromuscular control of walking in monkeys and humans, the spatial distribution of their functional maps may also have differences. However, unlike cats, monkeys and humans also share similarities such as signs of interlimb coupling which contribute to the diagonal footfall in monkeys^44^. We therefore posit that the distribution of the functional maps in humans is more similar to monkeys than that of the cats.

### Stimulation thresholds

A comparison of the distribution of the stimulation thresholds in monkeys (Fig. 5) with that of cats reported in the literature^20^ suggests that in both species, the largest proportion of the stimulation amplitudes are ≤10 µA (Fig. S5). This similarity in the stimulation thresholds between cats and monkeys is critical because it indicates that not only are the motor networks across species equally excitable, but also that functional movements can be evoked in non-human primates with current amplitudes that have been proven to be safe for ISMS^6,27^. The similarity in the distribution of the stimulation thresholds in cats and monkeys suggests a similar distribution may exist in humans as well.

### Kinematics and kinetics of the evoked movements

As observed in the functional maps (Fig. S1), ~45% of the mapped locations within the enlargement did not produce a motor response. This indicates that: *(i)* ISMS does not produce generalized activation of the ventral horn; instead, it produces targeted activation of movement-related neural networks; and *(ii)* targeting accuracy in ISMS is important. Similar to other neuroprostheses such as deep brain stimulation implants, targeting accuracy is necessary to achieve the best functional outcomes with ISMS. When proper targeting is achieved, the ISMS-evoked responses have the required characteristics for producing functional standing and walking. This is consistent with ISMS in other species^5,20,21^.

Movements produced by ISMS through single electrodes in this study were functionally highly relevant. The total ROM of the hindlimb joints produced by ISMS were similar to those reported for these joints during quadrupedal stepping on a treadmill. This suggests that by capitalizing on the functional connectivity within the lumbosacral cord, ISMS can produce movements with the ROMs needed for locomotion in rhesus monkeys.

ISMS was also able to evoke functional levels of torque, with knee extension torques reaching up to 91% of the levels evoked by supra-maximal nerve stimulation. This suggests that nearly all the motoneurons innervating the knee extensor muscles were recruited by ISMS through one stimulation site. Because the ISMS current does not directly spread throughout the ~16 mm extent of the quadriceps motoneuronal pools activation of the motoneurons therein was likely accomplished trans-synaptically through the activation of axons in passage around the electrode tip. In addition to amplifying force production, trans-synaptic activation of motoneurons results in the recruitment of motor units in a near normal physiological order^26^, produces graded increases in force^37,45^ and reduces the rate of muscle fatigue^18^.

In addition to the relatively large torques around a single joint, ISMS at some sites evoked extensor synergies in the ipsilateral leg that produced isometric forces up to 40% of the animal’s body weight. The strength of these synergistic movements further indicates the activation of functional networks that not only connect the motoneurons within a pool, but also connect motoneurons across pools innervating different muscles. Furthermore, the ability of ISMS to generate functional movements and torques in non-human primates suggests that this may also be the case in humans.

### Muscle activity

The evoked EMG activity patterns suggest that the gastrocnemius, biceps femoris posterior, and tibialis anterior muscles are commonly activated together by ISMS (Fig. 9). This is consistent with the evidence of strong heteronymous Ia afferent excitation^46^ and heteronymous recurrent inhibition^47^ between the gastrocnemius and biceps femoris muscles in humans. Similarly, coactivation of the gastrocnemius-biceps femoris pair is also reported for cats during unrestrained walking and trotting^48^. Antagonistic coactivation of the tibialis anterior and gastrocnemius muscles may serve to increase the stiffness and stability of the ankle joint and has also been reported in various phases of walking in humans^49^. The functional (Fig. 3) and EMG (Fig. 9) maps show common activation of the gastrocnemius, biceps femoris posterior, and tibialis anterior muscles during backward synergy movements as well.

The vastus and sartorius muscles also had a similar activation pattern in the rostral segments of the enlargement. Consistent with this observation, stimulation in a subset of these locations also evoked synergistic knee extension and hip flexion movements. Similarity of the activation pattern of the semimembranosus and gastrocnemius muscles in the caudal segments of the enlargement (L6–L7) is also consistent with the production of synergistic movements such as hip extension-ankle extension and extensor synergy.

A comparison of the segmental distribution of the EMG activity for the sartorius and tibialis anterior muscles (L3–L7 and L5–L7, respectively) with the anatomical map of their motoneuronal organization^10^ (L5–L6 and L4, respectively) shows a larger representation of these muscles across the functional map of lumbosacral enlargement. In contrast, the segmental distribution of EMG activity of the gastrocnemius muscles are more consistent with that of their motoneuronal map (L6–L7)^10^.

Collectively, these results suggest that similar to cats^4,5,20^, ISMS in a given site in the ventral horn of the spinal cord of monkeys not only activates the targeted motoneuronal pool, but also activates the functional networks, and ultimately the synergistic muscles, connecting relevant motoneuronal pools to each other^50^.

### Implications for clinical translation of ISMS implants

Recent studies of spinal-cord neuroprostheses have shown promising results for improving lower limb mobility after SCI. Implementation of intensive rehabilitation training paradigms involving epidural stimulation and treadmill training have demonstrated the potential of epidural stimulation for enhancing functional recovery, especially for people with incomplete SCI^51–53^. However, the functional benefits of these interventions have been limited for people with complete injuries^51,53^. By targeting the motor networks in the ventral horns of the spinal cord, ISMS can specifically produce various coordinated leg movements necessary for functional tasks such as walking. Preclinical studies have shown that ISMS can produce standing and walking even after a chronic complete SCI (transected spinal cord)^5^. Therefore, ISMS has the potential to restore walking for people with more severe SCIs than may be possible with epidural stimulation.

Knowledge of the functional organization of the motor interneuronal networks in the lumbosacral spinal cord of humans is essential for the clinical translation of ISMS implants. This includes knowledge about where in the spinal cord to place the implant for successful targeting of the leg movements required for functional standing and walking, and how to design the implant. Technical design considerations are: *(i)* the layout of the clinical implant (i.e., number of microelectrodes in the array, spacing between the microelectrodes, and targeting depth and length of the microelectrodes), and *(ii)* the specifications of the clinical microelectrode and stimulator that would safely deliver the current intensities required for producing functional movements.

Since functional mapping of the lumbosacral enlargement of the human spinal cord with a high spatial resolution, as in the present study, is not clinically feasible, and mapping with a low spatial resolution would require a large sample size to yield a complete functional map, we chose to study a non-human primate as the first step. The rhesus macaque monkey was chosen for this study as the closest available neurophysiological animal model to humans. The functional maps obtained for the monkey spinal cord along with the trends in the functional and anatomical organization of the motoneuronal pools in the discussed mammalian spinal cords can help predict the functional maps in humans.

Importantly, the similarity between the relative organization of the functional maps of the lumbar enlargement of the cat and monkey spinal cords suggests a similar relative organization for the functional map of the human lumbosacral spinal cord. However, differences exist between the spinal cord spatial and segmental distributions of the various movements in the functional maps of cats and monkeys. Assuming monkeys to be a closer animal model to humans (vs. cats), we hypothesize that the spatial and segmental distributions of various movements in the lumbar enlargement of the human spinal cord would be more similar to that of the monkeys. Along the path for clinical translation of ISMS, our team is planning to test this hypothesis in the upcoming intraoperative testing experiments in humans involving relatively coarse (low spatial resolution) electrophysiological mapping of the lumbar enlargement.

## Methods

All experiments were conducted in accordance to protocols approved by the Institutional Animal Care and Welfare Committee at the University of Washington (Seattle) and the University of Alberta (Edmonton).

### Surgery and the experimental setup

All surgical procedures and data collection were conducted under a continuous intravenous infusion of sodium pentobarbital anesthesia combined with fentanyl analgesia. The lumbar enlargement was exposed by laminectomy of the T12 to L2, L3, or L4 vertebrae in animals with 6, 7, or 8 lumbar vertebrae, respectively.

Prior to electrode implantation, the dura mater and arachnoid were opened and retracted with sutures placed in the paraspinal muscles. Four pedicle screws (3.5 mm diameter × 25 mm length, Medtronic Inc., Dublin, Ireland) were placed bilaterally in L1 and L3 pedicles, over which a spine-mounted stereotactic setup^31^ was assembled. The setup consisted of 2 frames and a platform with a micromanipulator that was used to place the microelectrode into the spinal cord^31^. The frames, which were secured to the pedicle screws, provided mechanical fixation over vertebral levels L1 to L3. The use of a spine-mounted setup was intended to reduce the relative movements between the spinal cord and the implanted microelectrode caused by breathing and limb movements, which reduced the risk of damage to the cord. To stabilize the spinal column further while allowing free movements in the hindlimbs, the animals were positioned in a custom-built suspension frame (Fig. 1). The pelvis was suspended with 2 pins at the iliac crest, while the spine was fixed with spinous process clamps at T12, L7 and S1.

In order to visualize the movements evoked by ISMS, the ankle was fitted with a string that was run over two pulleys and attached to a counter weight that balanced the weight of the leg (Fig. 1). Reflective markers were placed on the hip, knee, ankle, and metatarsophalangeal (MTP) joints, and on the distal phalanx of the 5^th^ digit. A video camera (120 fps, JVC, Yokohama, Japan) was used to capture movements of the leg in the sagittal plane.

EMG activity was recorded using a Ripple Grapevine neural interface system (sampling frequency: 2000 Hz; Ripple, Salt Lake City, UT, USA) through bipolar intramuscular electrodes placed in 8 hindlimb muscles.

Force measurements were obtained isometrically using a 150 lb-load cell (Interface Inc., Scottsdale, AZ, USA) at a sampling frequency of 1000 Hz. A custom-built adjustable stand was used to position the load cell perpendicularly to the axis of a given limb segment while blocking the movement. Hindlimb joints which were not involved in the ISMS-evoked movement were held in-place and fixed manually during the recording. For measurements of hip flexion and extension torque, the transducer was placed proximal to the knee joint against the anterior and posterior side of the thigh, respectively. For measurements of knee flexion and extension torque, the transducer was placed proximal to the ankle joint while fixing the hip joint manually. Similarly, for measurements of ankle flexion and extension torque, the transducer was placed at the midpoint of the foot, while fixing the proximal joints manually. For measurements of the evoked force for the extensor synergies, the transducer was placed at the midpoint of the foot plantar surface, with the iliac crest fixed bilaterally to the suspension frame. Other than the fixation at the iliac crest, all hindlimb joints were free to move. The moment arms were measured for all joints in each animal and were used to convert the measured forces to torque. Due to the complexity of synergistic movements, the measured forces are reported directly and were not converted to torque.

At the end of experiments, custom-made bipolar nerve cuffs were placed around the femoral nerve (in the femoral triangle) and the tibial nerve to measure isometric forces produced by the quadriceps and triceps surae (gastrocnemius and soleus) muscle groups, respectively, during supra-maximal nerve stimulation.

### Microelectrode insertion in the spinal cord

Platinum/iridium (Pt/Ir 90%/10%) microelectrodes 75 µm in diameter (Microprobes, Gaithersburg, MD, USA), were used for ISMS. Care was taken to advance the microelectrode tip to the ventral horn of the spinal cord while inserting the shaft of the electrode parallel to the minor axis of the spinal cord and without dimpling (compressing) its dorsal surface. Alignment of the insertion trajectory of the microelectrode was performed using an end-to-end alignment approach at the beginning of each experiment. This approach involved the use of two 2D bubble levels (5 mm in diameter, Level Developments Ltd., Chicago, IL, USA) over the dorsal surface of the spinal cord and over an L-shaped stylus held by the micromanipulator. The bubble levels were used to align the electrode holder perpendicularly to the surface of the spinal cord (Fig. S6).

In order to puncture the pia matter and avoid dimpling of the dorsal surface of the spinal cord during microelectrode insertion, a custom-made needle guide was used. The needle guide consisted of a 30-gauge needle, which could be lowered independently of the stimulating microelectrode. The microelectrode was fed through the lumen of the needle, and advanced into the cord after the needle punctured the pia.

The mapping protocol involved the insertion of the microelectrode into the spinal cord in steps of 2 mm × 0.5 mm in the rostrocaudal and mediolateral directions, respectively. This pre-planned mapping resolution was subject to change in cases where a dorsal vessel was in the way of the microelectrode. For each electrode track, stimuli were delivered at sites 0.5 mm apart dorsoventrally. The shallowest locations mapped were 2.5 mm from the dorsal surface of the cord.

### Electrical stimulation protocol

(a) Spinal cord stimulation: The ISMS protocol consisted of 0.5 s-train of biphasic, charge-balanced pulses, 200 µs-long and delivered at 50 Hz frequency. The stimulation amplitude ranged from 10 µA to a maximum of 300 µA. (b) Femoral and tibial nerve stimulation: 0.5 s-train of biphasic, charge-balanced pulses, 200 µs in width and 50 Hz frequency, with amplitudes ranging from 0.5 to 10 mA.

### Post-mortem spinal cord extraction

At the end of each experiment, animals were deeply anesthetized and perfused transcardially with a 4% formaldehyde solution for tissue fixation. The spine was then carefully dissected and the vertebral levels were identified based on the articulation with the last rib and lumbosacral transition. The laminectomy was expanded to visualize all nerve roots from the last thoracic level to S2 or S3. The fixed lumbosacral spinal cord with identified roots was then extracted and preserved in the formaldehyde solution for further processing. To reconstruct the mapped region of the spinal cord, cord segments were identified based on the entry zones of the dorsal rootlets. The boundaries of segment levels were marked by inserting 5 mm long glass tubes (175 µm in diameter, Wale Apparatus, Hellertown, USA) into the cord for identification in MRI images and histological sections (Fig. S7).

### Magnetic resonance imaging

The spinal cord samples were inserted in glass tubes filled with Fluorinert fluid (FC-770, Milipore Sigma, Darmstadt, Germany). The cords were imaged using a 3D gradient echo sequence with a resolution of 0.125 × 0.125 × 1 mm (Fig. S7). Imaging was performed on a 4.7 T Varian MR imaging system (Varian Inc., Palo Alto, CA, USA) using a 38 mm diameter volume coil employing the Litz design^54^ (Doty Scientific, Columbia, SC, USA).

### Tissue histological processing

The extracted spinal cord samples were cut in 50 µm cross-sections and stained using Mallory’s trichrome and neutral red Nissl stains for gross morphological analysis. Spinal cord sections were also analyzed to find electrode tracks from the mapping procedure (Fig. S7).

### Force, EMG and kinematic data analyses

EMG signals were filtered using a high-pass second order Butterworth filter with a corner frequency of 20 Hz to reduce motion artifacts. Kinematics, force and EMG recordings were analyzed using custom written programs in Matlab (version 2015a, MathWorks, Natick, MA, USA).

### Map creation

The functional map of the lumbosacral spinal cord was constructed primarily based on the movements that were evoked by ISMS. Three-dimensional coordinates of each of the mapped locations within the spinal cord were recorded using the coordinate system of the micromanipulator of the spinal stereotactic system. In order to link this external coordinate system with MR images and histological sections, a superficial suture was placed in the spinal cord at known rostrocaudal coordinates at the end of each experiment. For each of the animals, map creation involved the following steps: *(i)* Tracing and digitization of the outlines of the gray and white matter from MR images of the extracted spinal cords. *(ii)* Histological processing of the spinal cord tissue. Suture tracks were used to link the mapped coordinates in the external coordinate system (stereotactic setup) to true spinal cord geometries. Electrode insertion trajectories were initially assumed to be parallel with the minor axis of the spinal cord after the trajectory alignment step (Fig. S7). These insertion trajectories were confirmed or corrected in the reconstructed map based on the electrode tracks found in histological sections. *(iii)* Illustration of the responses for various movements with their corrected coordinates were superimposed on the digitized traces of the spinal cord gray and white matter.

### Spatial distribution of the functional and anatomical maps in cats

Functional^5,20,21^ and anatomical^35,39^ maps of the lumbosacral spinal cords of cats in the literature are presented only based on spinal cord segments and do not include a size estimate for each segment. In order to compare the spatial distribution of these maps, knowledge of the sizes of the segments in the cat spinal cord was necessary. As part of this study, 4 cat spinal cords (cord segments L1-S1) were extracted post-mortem after perfusion with 4% paraformaldehyde solution. Spinal cord segments were identified and marked with glass tubes as described earlier in ‘Post-mortem spinal cord extraction. For all spinal cords, the sizes of the marked cord segments were then measured with a ruler and used to create the spatial distribution maps shown in Fig. 11.

### Statistical analyses

The stimulation thresholds for evoking single- and multi-joint movements were compared using a t-test. The difference was considered significant for p < 0.05. Statistical analyses were performed using Microsoft Excel (version 16.19, Microsoft Corporation, Redmond, WA, USA).

## Supplementary information

Supplementary Information
