# Supplementary material for: Functional organization of motor networks in the lumbosacral spinal cord of non-human primates
Source: Sci Rep. 2019 Sep 19;9:13539. doi: 10.1038/s41598-019-49328-1 (PMC6753145; doi:10.1038/s41598-019-49328-1)
Supplement: Supplementary file 1 — Supplementary Information [file 41598_2019_49328_MOESM1_ESM.docx]

Supplementary Information for

**Functional organization of motor networks in the lumbosacral spinal cord of non-human primates**

Amirali Toossi, Dirk G. Everaert, Steve I. Perlmutter, Vivian K. Mushahwar

Vivian K. Mushahwar

Email: [vivian.mushahwar@ualberta.ca](mailto:xxxxx@xxxx.xxx)

**This PDF file includes:**

Figs. S1 to S7

**
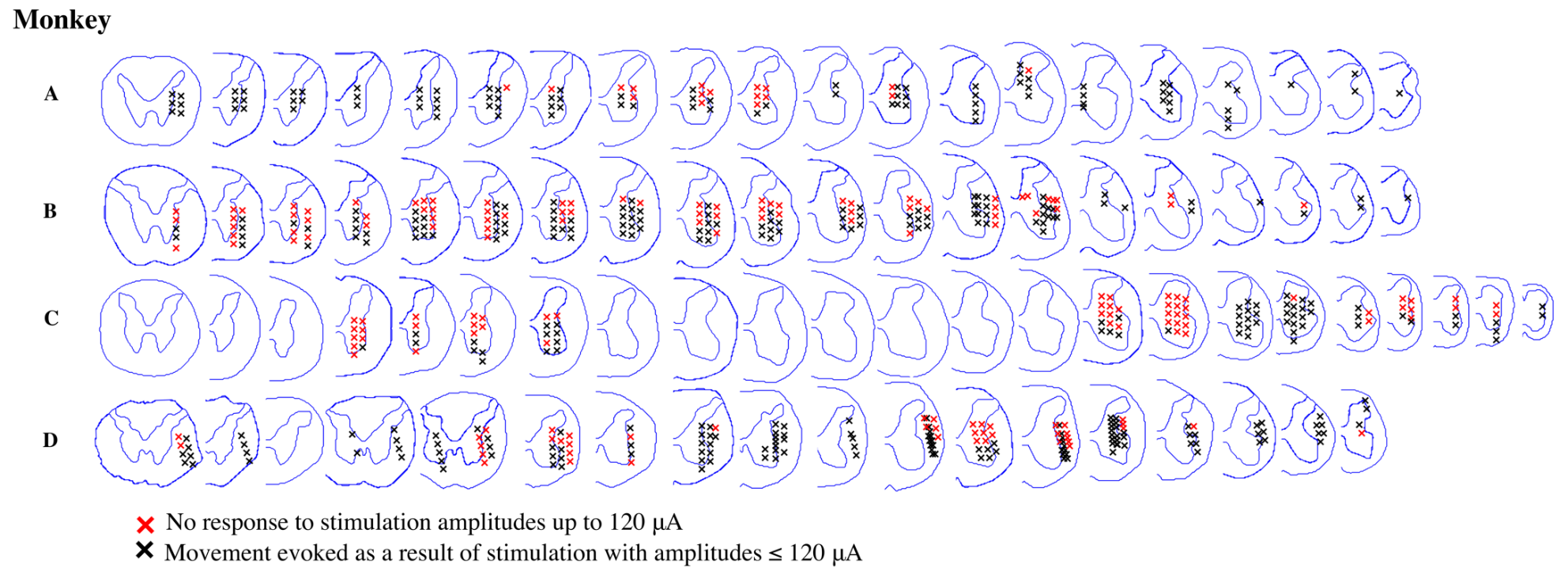
**

**Fig. S1.** All stimulated locations within the spinal cord across all animals. The cross-sections are sequential, separated by 2 mm. Spinal cord cross-sections of animal C, that do not show ‘x’s were not mapped.


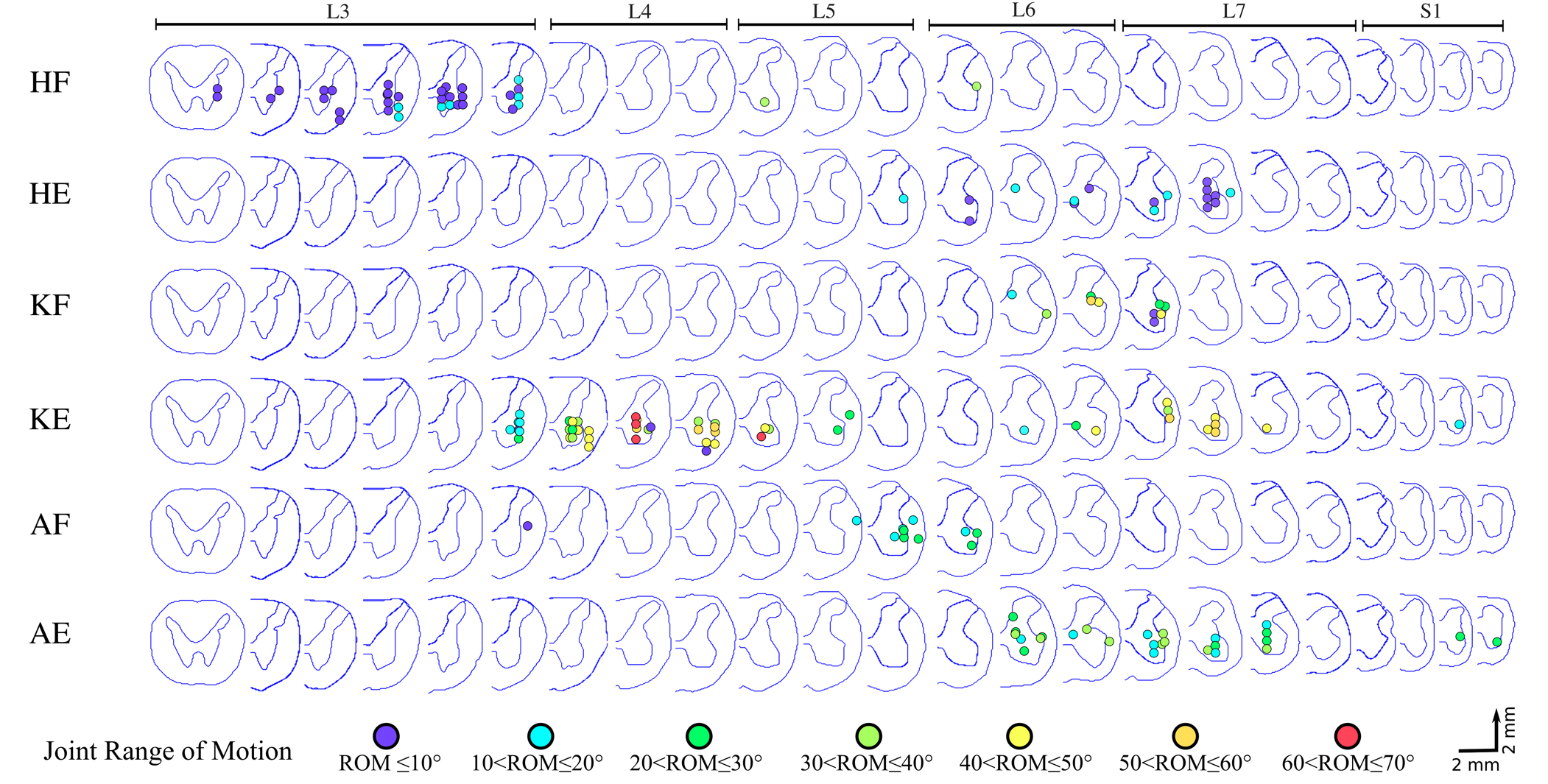


Fig. S2. Distribution of the ISMS-evoked range of motions in the hindlimb joints in all animals (n=4). Kinematic recordings were obtained from 40% of the sites with ISMS-evoked movements. Initial hip angle for the hip extension and flexion movements were 114.6° ± 3.2° and 112.6° ± 3.3° (mean ± standard error), respectively. Initial knee angle for the knee extension and flexion movements were 84.8° ± 3.86° and 102.9° ± 7.9° (mean ± standard error), respectively. Initial ankle angle for the ankle extension and flexion movements were 116.1° ± 3.9° 114.5° ± 5.4° (mean ± standard error), respectively. HF: Hip Flexion, HE: Hip Extension, KF: Knee Flexion, KE: Knee Extension, AF: Ankle Flexion, AE: Ankle Extension.


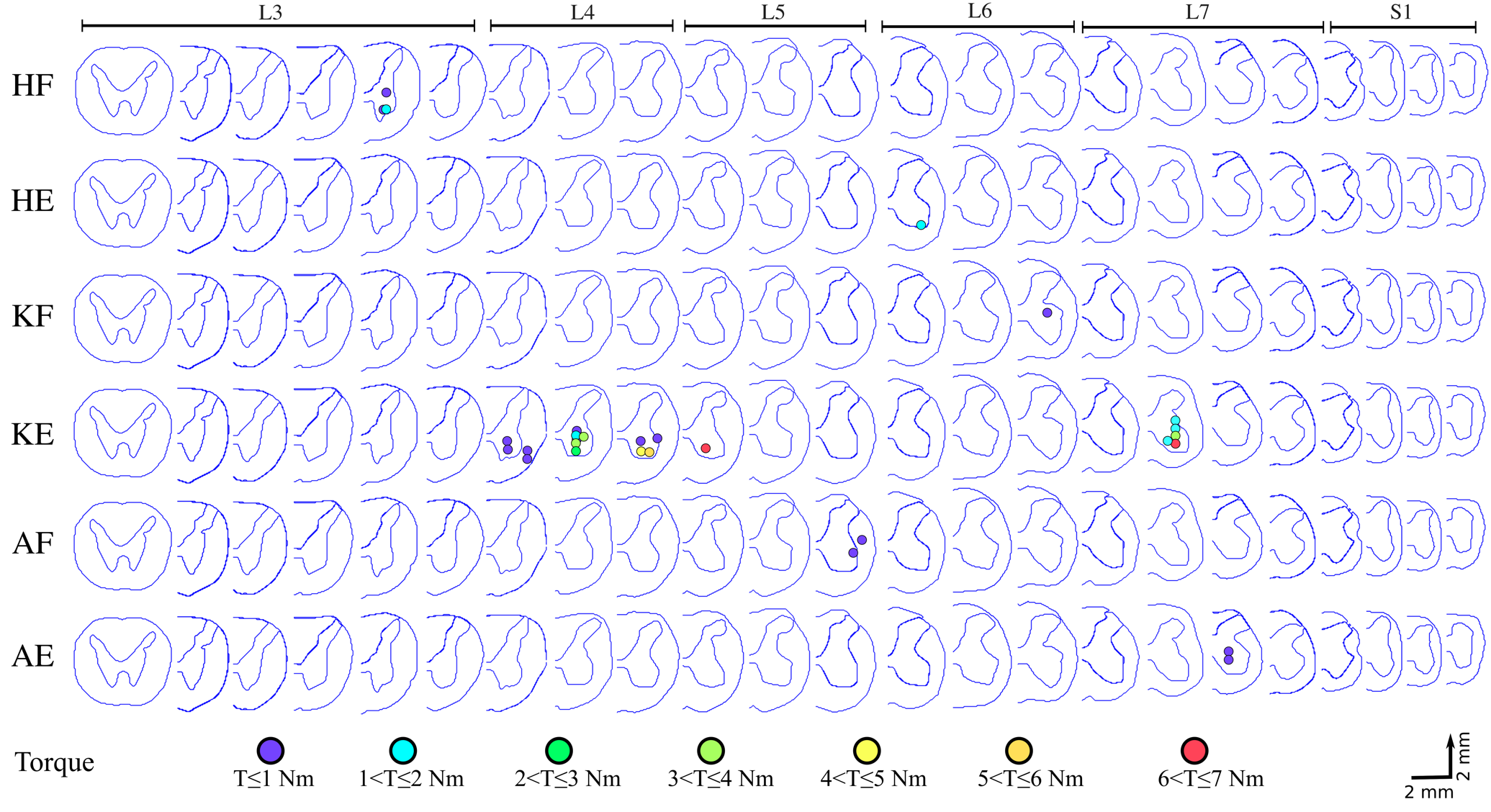


Fig. S3. Distribution of the ISMS-evoked joint torques in all animals (n=4). Torque recordings were obtained from 28 locations producing single-joint movements in response to ISMS. HF: Hip Flexion, HE: Hip Extension, KF: Knee Flexion, KE: Knee Extension, AF: Ankle Flexion, AE: Ankle Extension.


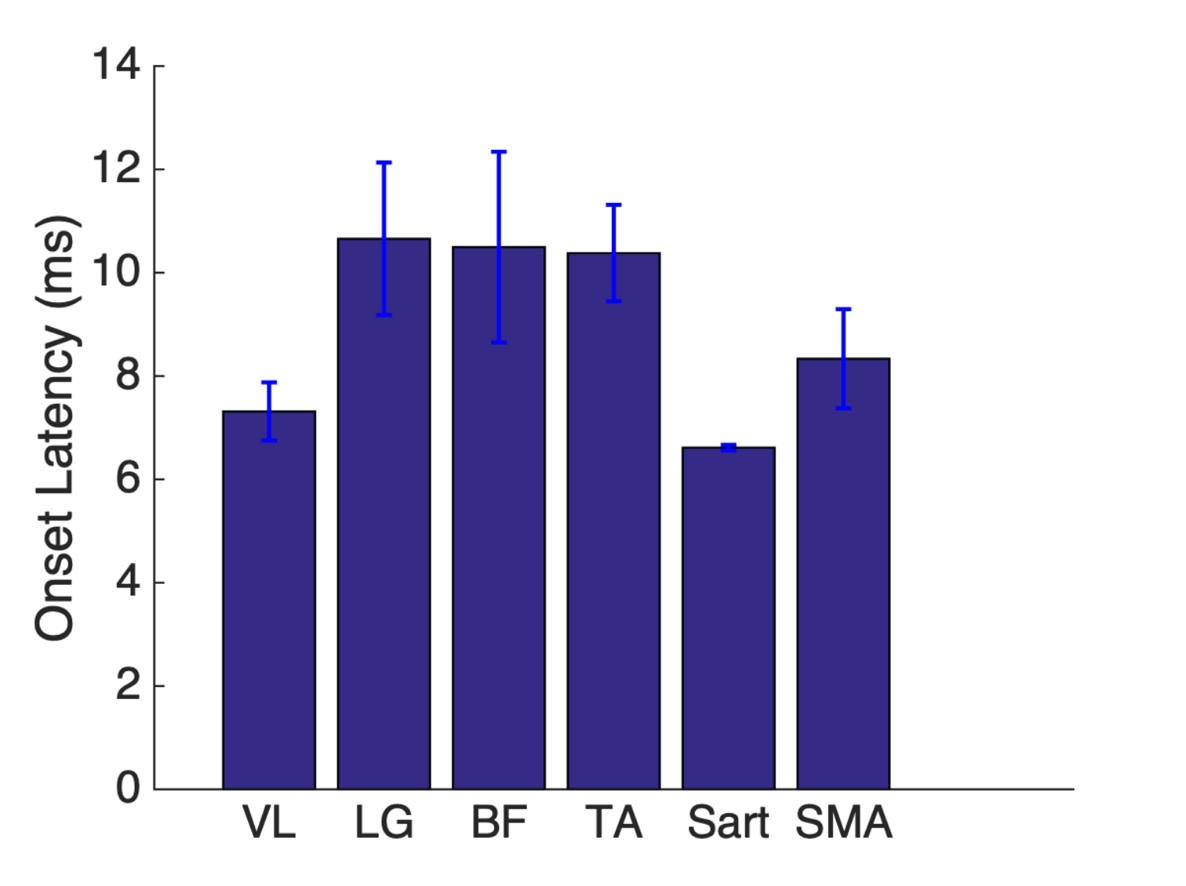


**Fig. S4.** Onset latencies of EMG responses. EMG onset latencies measured in 3 animals (A, C, and D). SMA: Semimembranous anterior; Sart: Sartorius; VL: Vastus Lateralis; TA: Tibialis Anterior BF: Biceps Femoris; LG: Lateral Gastrocnemius.


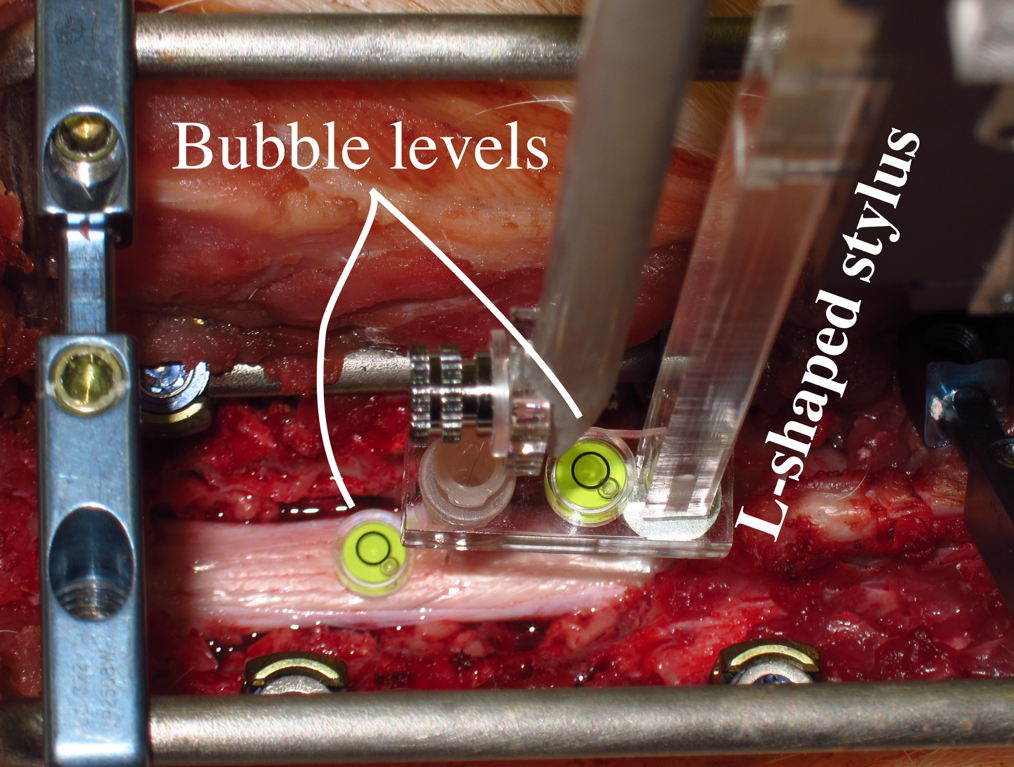


Fig. S5. Microelectrode alignment using bubble levels

**
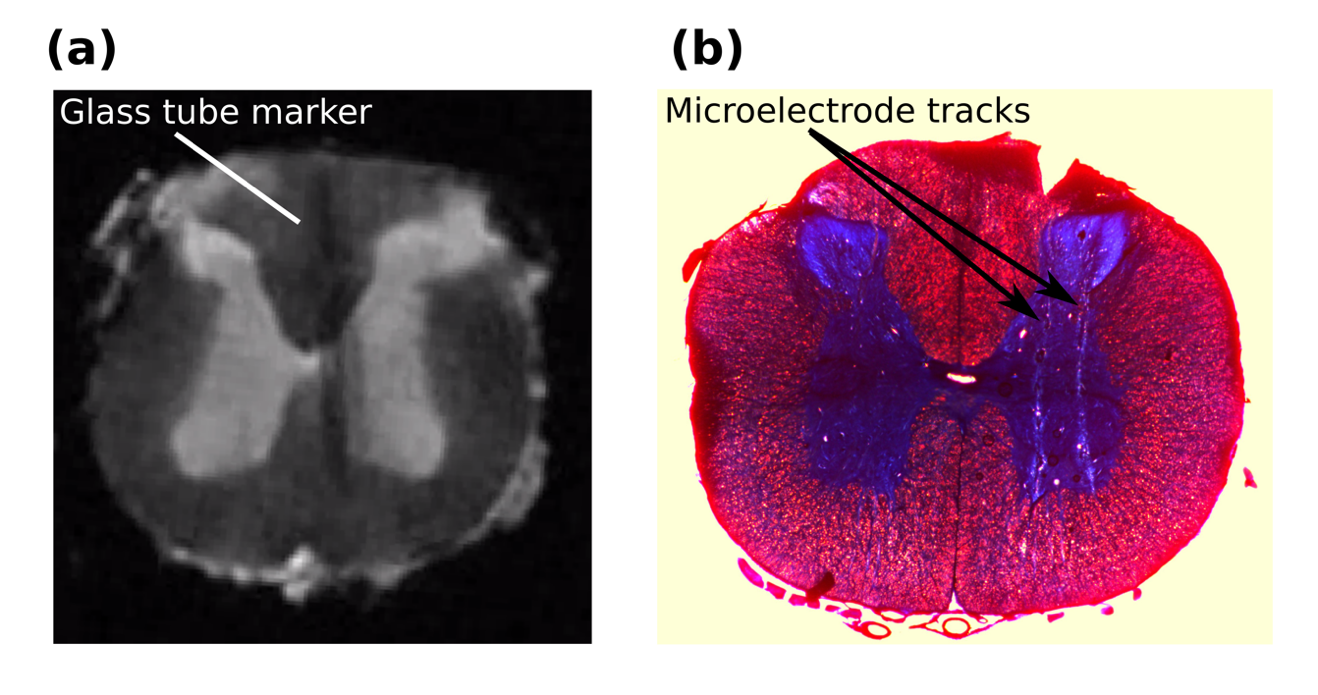
**

Fig. S6. (a) An example of an MR image of the spinal cord (animal B) in the transverse plane. The marked black line is the imaging artifact from a glass tube marker inserted into the spinal cord at the boundary of L4-L5 spinal cord segments. (b) Microscopic image of a 50 µm thick cross-section of the spinal cord (animal C – spinal cord level L3) showing two electrode tracks. Tissue was stained with the Mallory’s trichrome stain.


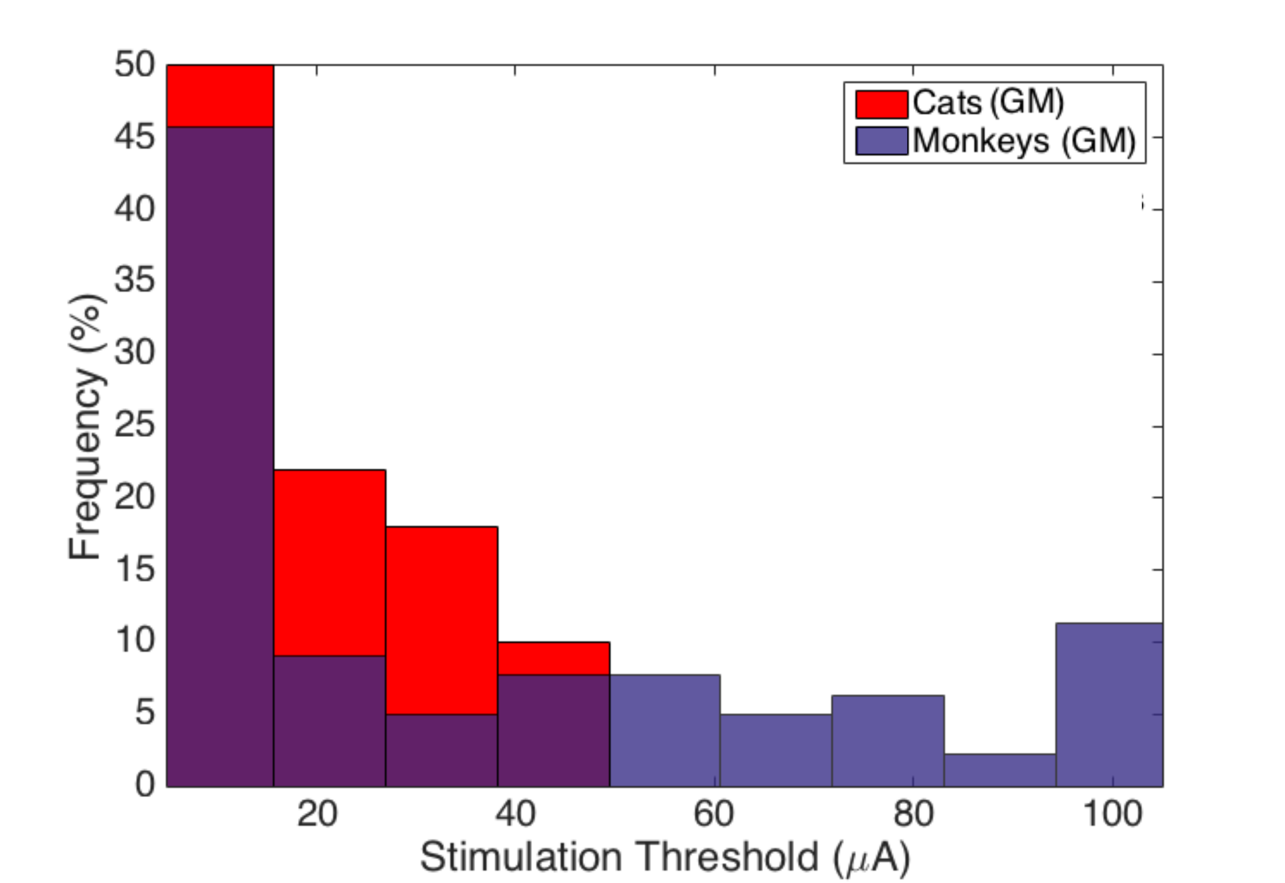


Fig. S7. The distribution of stimulation thresholds in cats and monkeys. The stimulation thresholds in rhesus monkeys were defined as the minimal amplitude (up to 120 µA) required for producing a visible leg movement or twitch. The stimulation thresholds in cats were based on the minimal stimulation amplitude (up to 40 µA) needed to produce a detectable isometric force or EMG signal in targeted muscles. Stimulation thresholds in cats represent measurements for the quadriceps, triceps surae and tibialis anterior muscles (n=6 animals /muscle)^20^ and stimulation thresholds in monkeys represent measurements from all of the evoked movements by ISMS in the gray matter of the lumbar enlargement (n=4 animals, total of 221 locations). Data from cats were adapted from Mushahwar and Horch.^20^
